# Forecast analysis of any opportunistic infection among HIV positive individuals on antiretroviral therapy in Uganda

**DOI:** 10.1186/s12889-016-3455-5

**Published:** 2016-08-11

**Authors:** John Rubaihayo, Nazarius M. Tumwesigye, Joseph Konde-Lule, Fredrick Makumbi

**Affiliations:** 1 Department of Epidemiology and Biostatistics, School of Public Health, College of Health Sciences, Makerere University, Kampala, Uganda; 2 Department of Public Health, School of Health Sciences, Mountains of the Moon University, P.O.Box 837, Fort Portal, Uganda

**Keywords:** HIV/AIDS, Opportunistic infections, Antiretroviral therapy, Prevalence, ARIMA, Forecast, TASO, Uganda

## Abstract

**Background:**

Predicting future prevalence of any opportunistic infection (OI) among persons infected with the human immunodeficiency virus (HIV) on highly active antiretroviral therapy (HAART) in resource poor settings is important for proper planning, advocacy and resource allocation. We conducted a study to forecast 5-years prevalence of any OI among HIV-infected individuals on HAART in Uganda.

**Methods:**

Monthly observational data collected over a 10-years period (2004–2013) by the AIDS support organization (TASO) in Uganda were used to forecast 5-years annual prevalence of any OI covering the period 2014–2018. The OIs considered include 14 AIDS-defining OIs, two non-AIDS defining OIs (malaria & geohelminths) and HIV-associated Kaposi’s sarcoma. Box-Jenkins autoregressive integrated moving average (ARIMA) forecasting methodology was used.

**Results:**

Between 2004 and 2013, a total of 36,133 HIV patients were enrolled on HAART of which two thirds (66 %) were female. Mean annual prevalence for any OI in 2004 was 57.6 % and in 2013 was 27.5 % (X^2^_trend_ = 122, b = −0.0283, *p* <0.0001). ARIMA (1, 1, 1) model was the most parsimonious and best fit for the data. The forecasted mean annual prevalence of any OI was 26.1 % (95 % CI 21.1–31.0 %) in 2014 and 15.3 % (95 % CI 10.4–20.3 %) in 2018.

**Conclusions:**

While the prevalence of any OI among HIV positive individuals on HAART in Uganda is expected to decrease overall, it’s unlikely that OIs will be completely eliminated in the foreseeable future. There is therefore need for continued efforts in prevention and control of opportunistic infections in all HIV/AIDS care programmes in these settings.

**Electronic supplementary material:**

The online version of this article (doi:10.1186/s12889-016-3455-5) contains supplementary material, which is available to authorized users.

## Background

Since the introduction of highly active antiretroviral therapy (HAART) in resource poor settings, there has been tremendous reduction in the incidence and overall prevalence OIs among persons living with HIV/AIDS [1]. In 2014, UNAIDS came up with very ambitious programme code named “90-90-90” meaning 90 % of all the people living with HIV to be diagnosed, 90 % of all diagnosed to receive ART and 90 % of all on ART to have a fully suppressed viral load by 2020 [2]. Basing on these targets and past achievements, there is global consensus that the HIV/AIDS epidemic could be halted by 2030 if the planned actions are fully implemented [3]. These targets have been adopted by many developing countries including Uganda and form the basis for national policy and strategic planning. Uganda 5 year National HIV strategic plan (2015/16-2019/2020) (NSP) targets to reduce HIV-associated morbidity and mortality by 70 % and maintain 90 % viral suppression through a strategy of increased HAART coverage, early HAART initiation and improved retention in HIV care [4, 5].

HAART rollout in public funded HIV/AIDS care facilities in Uganda started in 2004 as part of the Global HAART roll out strategy [6]. The Uganda government employed a public-private sector partnership strategy to accelerate the HAART rollout exercise. By the end of 2014, a cumulative total of 750,896 HIV positive patients out of the estimated 1.6 million HIV positive individuals in Uganda (50 %) had access to HAART [4]. However, despite these impressive gains, there are still disparities in HAART usage by geographical area, sex and age [5] and the effect of increasing HAART coverage on frequency and patterns of OIs among HIV positive individuals over time and in different geographical areas is not well known.

Effective strategies for prevention of opportunistic infections and mortality associated with HIV/AIDS is of great importance given the high cost and uncertainty surrounding sustainability of lifelong ART in resource poor settings. There is therefore need to plan ahead of time in order to make current treatment/preventive interventions more effective. An accurate morbidity prediction of any OI among HIV-infected patients on HAART provides a good scientific basis for planning and formulating appropriate control or preventive policies.

The Box-Jenkins autoregressive integrated moving average (ARIMA) model is the most widely used method in forecasting of infectious diseases [7–14]. ARIMA fits univariate models for a time series where time dependent disturbances are allowed to follow a linear autoregressive moving average specification [15, 16]. Univariate ARIMA forecasting is a procedure for computing a forecast based on past and present values of a given series possibly augmented with a function of time (e.g. a linear trend) [16]. However, univariate ARIMA forecasting is appropriate where there is large number of series to forecast and the time series are stationary (i.e. constant variance or rate of change). ARIMA models have been used successfully to monitor and forecast different infectious diseases including new annual HIV infections in Korea [17], monthly dengue fever cases in Thailand [9], monthly dengue incidence in Brazil [12], hantavirus outbreak in Chile [13], SARs outbreak in Singapore [11], hemorrhagic fever outbreak in China [10], monthly malaria cases in Burundi [14], monthly TB morbidity in China [7] and monthly mortality rate in under-five years old children in Iran [18].

However to the best of our knowledge, there has not been any published work on prediction of OIs associated with HIV/AIDS among HIV positive patients on HAART in Uganda. The purpose of the current study therefore was to model and forecast the prevalence of any OI among HIV-infected individuals on highly active antiretroviral therapy (HAART) in Uganda.

## Methods

### Study settings

Data for this study was obtained from the AIDS Support Organization (TASO) in Uganda. TASO is the oldest and largest non-governmental organization (NGO) providing HIV/AIDS care and treatment in Uganda and sub-Saharan Africa [19]. TASO started in 1987 and has since established 11 HIV clinics spread across Uganda serving mainly the rural population [20]. TASO HIV/AIDS care and treatment programme offers a wide range of services include HIV testing and counseling, antiretroviral therapy, primary and secondary prophylaxis, OI diagnosis and treatment, home based care and psycho-social support.

With close to 3 decades of experience in HIV/AIDS care and treatment, TASO provided a good opportunity for assessing HAART effect on trends of opportunistic infections in real life programmatic settings in which HAART roll out is taking place in sub-Saharan Africa [21]. Initially, access to HAART was based on the Ugandan ministry of Health and the World Health Organization (WHO) 2006 guide lines as indicated above [22, 23]. However, after 2010 access to HAART was expanded to include HIV patients with CD4 cell count ≤350 or WHO clinical stage3 or 4 irrespective of CD4 cell count as was recommended by Ugandan Ministry of Health and WHO/UNAIDS [24, 25]. By end of 2013, a total of 91,218 were actively in care of which 58,051 clients(64 %) were on HAART and 87,903(96.4 %) were on cotrimoxazole/dapson prophylaxis throughout the 11 HIV clinics across Uganda [20]. All services are free of charge including anti-retroviral drugs (ARVs) for those who are eligible [21].

### Sampling and sample size

**Fig. 1:**
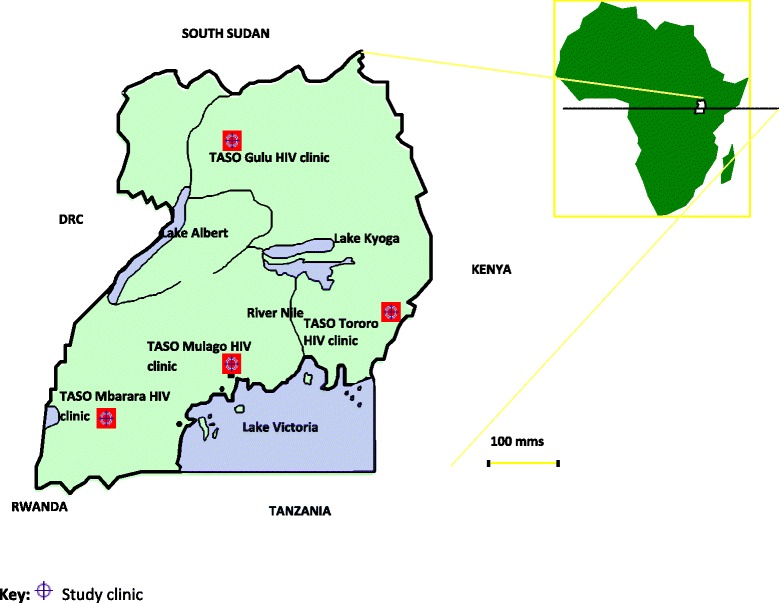
Map of Uganda showing the location of study sites

Four TASO HIV clinics were purposively selected basing on volume, quality of data and geographical representation. The HIV clinics selected were TASO Mulago HIV clinic in central Uganda, TASO Mbarara HIV clinic in south-western Uganda, TASO Tororo HIV clinic in Eastern Uganda and TASO Gulu HIV clinic in Northern Uganda (Fig. 1). All HIV positive adults (15 years and above) who attended at least once at the selected HIV clinics in the period from 1^st^ January 2004 to 31^st^December 2013 were included in the study.

### Data collection

Data collection procedure was according to an established protocol across all TASO HIV clinics. In brief, each client is expected to attend the clinic at least once a month. At each clinic visit, data per client is collected on a standardized case report form (CRF) detailing the client’s demographic characteristics, clinical condition, medical history, prophylaxis use, OI diagnosis and treatment given. The information was then compiled and entered into the TASO electronic data base using EPIINFO vs3 in Access format. Data on 14 AIDS-defining OIs and 2 non-AIDS defining OIs (malaria & geohelminths) plus HIV-associated Kaposi’s sarcoma were compiled and handed over for analysis.

### Statistical analysis

To establish whether there is a trend, data on prevalence of any OI was summarized by month and calendar year. Then a monthly time plot was drawn. Monthly prevalence was calculated as a proportion of clients diagnosed with any OI in a given month. Mean annual prevalence was calculated as an average of the 12 monthly prevalences in a year. To examine long term trends in monthly prevalence, the moving average smoothing technique was used to filter out short-term fluctuations and random variation within the monthly series [26]. The moving average smoothing technique achieves this by replacing each element of the time series by *n* neighbouring elements, where n is the width of the smoothing window. A centered moving average including three observations before and two observations after the current observation inclusive was used.

To forecast, the Box-Jenkins ARIMA methodology [27] was used. This method involves an interactive procedure including: model identification, evaluation and diagnostic checking before forecasting. Since ARIMA requires a stationary process, the Augmented Dickey-Fuller (ADF) unit-root test was used to test for stationarity of the monthly series. Non-stationary series were transformed by first order differencing to stabilize the variance. Autocorrelation function (ACF) and partial autocorrelation function (PACF) plots were used to determine possible values for the autoregressive and moving average orders. Akaike information criterion (AIC) and Bayesian information criterion (BIC) were used to identify the most parsimonious model. Parameter estimation was by maximum likelihood method. Diagnostic checks involved plotting ACF and PACF for autocorrelation structure and the Portmanteau test for white noise in the model residuals.

In order to evaluate the model forecast accuracy, data were split into two groups: one for the fitting process (2004–2010) and the other for validation (2011–2013). Forecast accuracy was assessed by computing the mean absolute percentage error (MAPE) [16]. Finally, the fitted ARIMA model was used to forecast 5 year mean prevalence of any OI among HIV positive patients on HAART for the period 2014–2018. Root mean squared error (RMSE) was used to estimate lower and upper forecast limits [28]. All analyses were conducted using Stata 13 (Stata Corp, TX) with *p* <0.05 considered significant.

## Results

Between 2004 and 2013, a total of 36,133 HIV patients were enrolled on HAART of which two thirds (66 %) were female with a median age of 33 year (IQR, 27–40) (Table 1). In the planning data (2004–2010), it was observed that mean annual any OI prevalence reduced from 56.62 % in 2004 to 36.61 % in 2010. While in the validation data (2011–2013) mean annual any OI prevalence reduced from 35.89 % in 2011 to 27.53 % in 2013 (Table 2).

**Table 1:** Baseline characteristics of study participants who were started on HAART in the period between 2004 and 2013

Variable	Total	Female	Male	*p*-value
Gender (36,133), *n*(%)				
Female	23,767(66)	-	-	-
Male	12,366(34)			
Median age(36,120), *n*(IQR)	33(27–40)	32(26–39)	36(29–43)	<0.0001
Age (36,120), *n*(%)				
<35 years	19,338 (54)	13,894 (58)	5444 (44)	<0.0001
>=35 years	16,782 (46)	9863 (42)	6919 (56)	
Location (36,133), *n*(%)				
Eastern	9793(27)	6271 (26)	3522(28)	<0.0001
Central	9493(26)	6573(28)	2920(24)	
Western	8576(24)	5426(23)	3150(25)	
Northern	8271(23)	5497(23)	2774(23)	
Education (*n* = 34,337), *n*(%)				
Primary/none	26,331(77)	18,165(81)	8166(69)	<0.0001
>=Secondary	8006(23)	4342(19)	3664(31)	
Marital status (*n* = 34,338), *n*(%)				
Single/never married	2148(6)	1260(6)	888(7)	<0.0001
Married	17,187(50)	9523(42)	7664(65)	
Divorced	5778(17)	4342(19)	1436(12)	
Widowed	6932(20)	6118(27)	814(7)	
others	2293(7)	1254(6)	1039(9)	
Occupation (*n* = 33,454), *n*(%)				
Paid employee	5731(17)	2611(12)	3120(27)	<0.0001
Self employed	12,157(36)	9193(42)	2964(26)	
Subsistence farmer	13,283(40)	8855(40)	4428(38)	
others	2283(7)	1196(6)	1087(9)	
WHO clinical stage (*n* = 36, 133), *n*(%)				
I&II	16,535 (28.5)	11,030 (28.5)	5505 (28.6)	0.773
III&IV	41,410 (71.5)	27,675 (71.5)	13,735 (71.4)	
Weight (23,848), *n*(%)				
<55 years	11,853(50)	8156(52)	3697(45)	<0.0001
>=55 years	11,995(50)	7466(48)	3697(55)	
ART regimes				
Stavudine	3926(11)	2743(11)	1183(10)	
Zidovudine	18,142(50)	11,881(50)	6261(50)	<0.0001
Tenofovir	9931(28)	6397(27)	3534(29)	
Other	4134(11)	2746(12)	1388(11)	
Year of enrolment on HAART				
2004–2008	12,433(34)	8439(36)	3994(32)	<0.0001
2009–2013	23,700(66)	15,328(64)	8372(68)

**Table 2:** Mean annual prevalence of any OI (2004–2013)

Visit year	Mean annual prevalence	Standard deviation
2004	57.62	6.36
2005	45.01	3.89
2006	42.12	2.29
2007	41.54	1.85
2008	39.06	3.67
2009	37.69	3.84
2010	36.61	2.25
2011	35.89	2.83
2012	34.16	3.52
2013	27.53	2.15

**Fig. 2:**
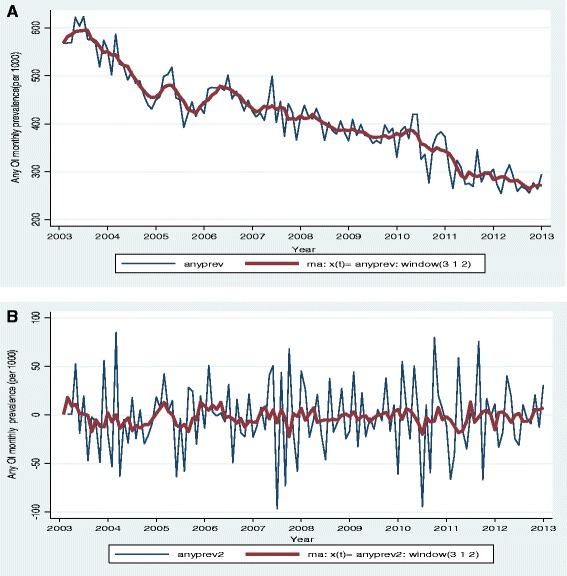
Plot showing (**a**) the original and smoothed any OI monthly prevalence series and (**b**) first order differenced any OI monthly prevalence series (2004–2013)

A time plot of the monthly OI prevalence trends shows several minor peaks along the series (Fig. 2). No seasonal or periodic components were clearly seen in the plot but the smoothed series generally depict a decreasing trend (Z statistic = −10.23, *p* <0.0001, nptrend) (Fig. 2). The Augmented Dickey-Fuller (ADF) test shows that the original monthly series had a unit root (z (t) = −2.353, *p* = 0.1556, lags = 20) suggesting that it’s not stationary. However after the first differencing the ADF test rejected the null hypothesis of a unit root in the series (z (t) = −16.7, *p* <0.001, lags = 20) implying that the differenced monthly series were stationary (Fig. 2). All further statistical procedures were performed on the stationary series.

**Fig. 3:**
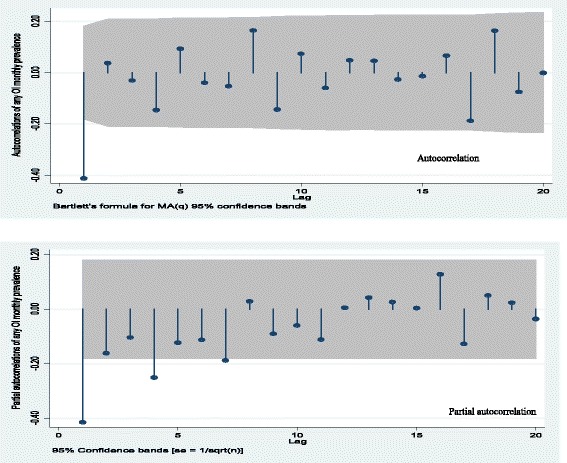
Plot of autocorrelation function (ACF) and partial autocorrelation function (PACF) for the first order differenced any OI monthly series

Model identification started with autocorrelation analysis. Plots of autocorrelation function (ACF) and partial autocorrelation function (PACF) (Fig. 3) showed only the first lag of the ACF was significant (i.e. laying outside the grey 95 % CI band). It was also observed that the first few lags of PACF were decaying with time. Based on the autocorrelation structure, several potential models were identified.

Using the Akaike information criteria (AIC) and Bayesian information criteria (BIC), several first order differenced models were evaluated. ARIMA (1, 1, 1), ARIMA (1, 1, 2) and ARIMA (2,1,1) were initially selected basing on their low AIC and BIC values (Table 3). Parameter estimation for ARIMA (1, 1, 1), showed that AR (1) coefficient of 0.23 and MA (1) coefficient of −0.86 were significant (*p* <0.05) (Table 4).

**Table 3:** AIC and BIC model selection criteria

Model	AIC	BIC	Rank
ARIMA (1,1,1)	870	881	1
ARIMA (1,2,1)	870	882	2
ARIMA (1,2,3)	870	884	3
ARIMA (1 1 3)	870	884	3
ARIMA (2,2,1)	870	884	3
ARIMA (2 1 1)	870	884	3
ARIMA (3, 2, 4)	870	889	4
ARIMA (1,2,2)	871	885	5
ARIMA (1 1 2)	871	885	5
ARIMA (3,2,3)	871	893	6
ARIMA (3 1 3)	871	893	6
ARIMA (2,2,2)	872	889	7
ARIMA (2 1 2)	872	889	7
ARIMA (3,2,1)	872	889	7
ARIMA (3 1 1)	872	889	7
ARIMA (4 1 3)	872	894	8
ARIMA (2 1 4)	873	895	9

**Table 4:** Estimation of model parameters for any OI

Model	Model parameter	Coefficient	*p*-value
Arima(1,1,1)	constant	−0.26	<0.0001
	AR(1)	0.28	0.008
	MA(1)	−0.88	<0.0001
	Sigma	3.2	<0.0001
Arima(1,1,2)	constant	−0.23	<0.0001
	AR(1)	0.73	<0.0001
	MA(1)	−1.41	0.999
	MA(2)	−0.41	0.999
	Sigma	2.99	0.499
Arima(2,1,1)	constant	−0.23	<0.0001
	AR(1)	0.32	<0.0001
	AR(2)	0.19	0.045
	MA(1)	−1.0	0.981
	Sigma	2.98	0.481

**Fig. 4:**
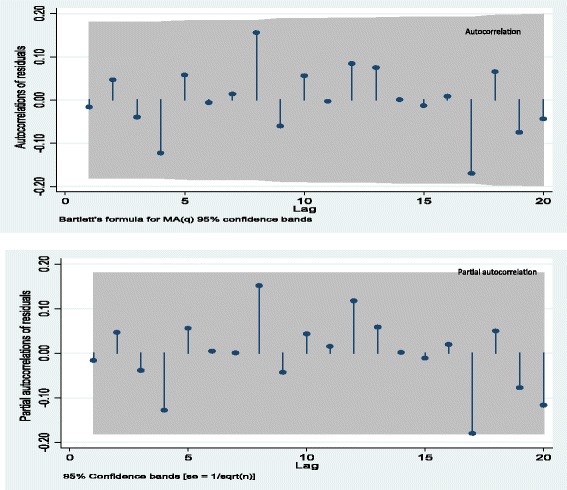
Plot of residuals autocorrelation function (ACF) and partial autocorrelation (PACF) for the fitted model to forecast any OI prevalence among HIV positive individuals on HAART

**Fig. 5:**
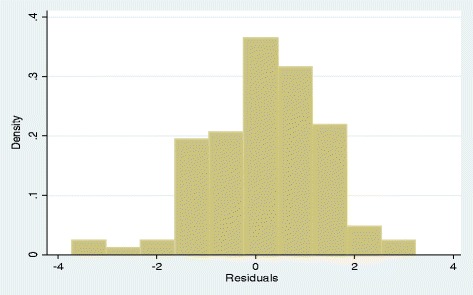
Histogram of the residuals

Plots of autocorrelation function (ACF) and partial autocorrelation (PACF) of the model residuals showed that ACFs and PACFs were not significantly different from zero (white noise) (Fig. 4). The portmanteau Q-test (Q_15_ = 8.20, *p* = 0.9153) was also in favour of the null hypothesis of no autocorrelation in the residuals. A plot of the residual histogram showed no volatility clustering and so assumed the residuals were homoscedastic (Fig. 5). The skewness-kurtosis test (X^2^ = 3.42, *p* = 0.154) was consistent with the normality assumption of the model residuals (Fig. 4.13).

Average annual prevalence rates declined from 58 % in 2004 when HAART was first rolled out to 28 % in 2013. The model estimated a decline in OI rate of −0.028 per year (95 % CI −0.034 to −0.023) or −0.0023 per month (95 % CI: −0.0028 to −0.0019).

**Fig. 6:**
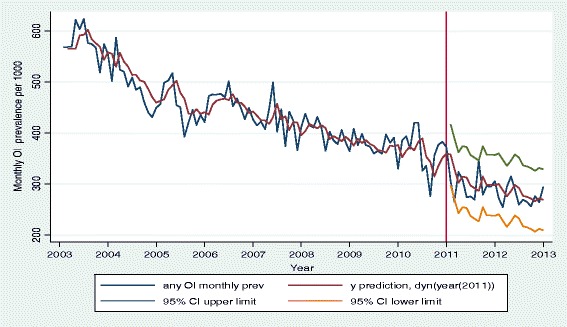
Plot of observed and predicted values for any OI among HIV positive patients on HAART from the fitted ARIMA (1, 1, 1) model with their corresponding 95 % confidence limits

**Fig. 7:**
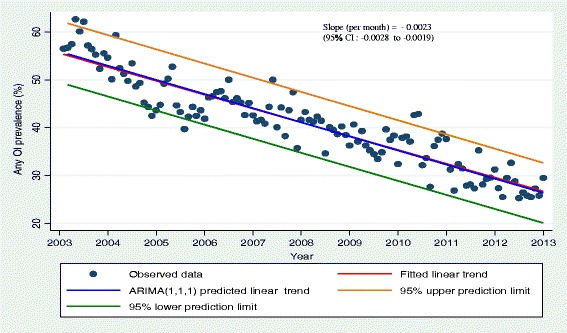
Scatter plot of observed data with fitted linear trend and ARIMA (1,1,1) predicted trend with 95 % confidence intervals

A plot of the observed and predicted values of any OI matched reasonably well (Fig. 6). A t-test of the observed and predicted mean annual any OI prevalence showed no significant difference (*p* >0.05) (Table 5). The predicted linear trend from the model and the fitted linear trend to the observed data had the same slope = −0.0023 (95 % CI: −0.0028 to −0.0019) (Fig. 7).

**Table 5:** t-test of the difference between observed and predicted mean annual any OI prevalence in the validation period (2011–2013)

Year	Observed		Predicted		t-test
	Mean annual any OI prevalence	SD	Mean annual any OI prevalence	SD	
2010	36.61	2.25	36.39	0.923	t = 1.5421, *p* = 0.151
2011	35.89	2.83	36.12	1.501	t = −0.620, *p* = 0.547
2012	34.16	3.52	34.21	1.404	t = −0.035, *p* = 0.973
2013	27.53	2.15	27.84	1.601	t = −0.519, *p* = 0.614

The forecasted mean annual prevalence of any OI was 26.1 % (95 % CI 21.1–31.0 %) in 2014 and 15.3 % (95 % CI 10.4–20.3 %) in 2018 (Table 6). The prediction error estimated in terms of mean absolute percentage error (MAPE) was 8.2 % (SD = 6.0).

**Table 6:** One-step ahead forecast of mean annual prevalence of any OI among HIV positive patients on HAART (2014–2018)

Time	Predicted value (per 100)	95 % Confidence limits	
		Lower predicted limit	Upper predicted limit
2014	26.1	21.1	31.0
2015	23.3	18.3	28.3
2016	20.7	15.7	25.6
2017	18.0	13.0	23.0
2018	15.3	10.4	20.3

## Discussion

ARIMA models have been widely used to predict morbidity and mortality due to infectious diseases with fairly good accuracy [7, 9, 10, 12, 17]. Early warning based on forecasts is very important for advocacy and resource allocation. For example ARIMA models have been successfully used to monitor and forecast new annual HIV infections in Korea [17], monthly dengue fever cases in Thailand [9], monthly dengue incidence in Brazil [12], hantavirus outbreak in Chile [13], SARs outbreak in Singapore [11], hemorrhagic fever outbreak in China [10], monthly malaria cases in Burundi [14], monthly TB morbidity in China [7] and monthly mortality rate in under-five years old children in Iran [18]. Yu et al. [17] used observation data for the period 1985–2012 to forecast new HIV infections for the period 2013–2017. ARIMA models can take into account changing trends, periodic changes and random disturbances in the time series [7].

This study established that ARIMA (1,1,1) can be used to predict monthly OI morbidity among HIV positive patients on HAART in Uganda. The fitted model suggests the need for first-order differencing of the series to remove a stochastic decreasing trend, then a first order autoregressive (1) term and a first order moving average (1) term to cater for serial correlation in the data. This means that prevalence of any OI each month is directly influenced by the presence of any OI in the preceding month and the prediction errors of the current and preceding month. The model can also be used to estimate the cost of OIs prevention/treatment in the next 5 years of HAART after 2013 as well as informing policy, planning and advocacy for increased resource allocation towards HIV/AIDS care in Uganda.

In this study, the forecasted prevalence of any OI was 15.3 % in 2018 which show that though the prevalence of any OI is likely to reduce but will not vanish as was reported in previous studies elsewhere [29]. Available reports suggest that OIs are likely to remain an important aspect of care even in the era of HAART especially in low income countries due to several factors including late HIV diagnosis, sub-optimal HAART use, poor adherence, dug resistance, poverty, poor nutrition, high exposure to infectious agents, just to mention a few [29–35]. Early warning based on forecasts is very important for future planning and rational resource allocation. There is therefore need for more efforts in prevention and control of opportunistic infections in all HIV/AIDS care programmes in these settings.

In the current study, results show that the ARIMA (1,1,1) model gives a good prediction of OIs prevalence in the period January 2014 - December 2018 (60 months) within 8 % margin of error i.e. MAPE = 8.2. The prediction error was close to that found in another previous study that used ARIMA (3,1,4) model to forecast dengue incidence in North-Eastern Thailand with a margin of error of 7 % (i.e. MAPE = 7.0) [36]. The difference could be attribute to the fact that our predictions covered a slightly longer period of 60 months compared to 40 months in the previous study.

One of the limitation of this study was that the ARIMA model was fitted with a constant term (1^st^ order differences) hence forcing the assumption that past trends (a reduction of 0.2 % in OI prevalence per month) will be maintained over time. This could only be possible if the current OI interventions are maintained and all other factors remain constant which may not be the case. Future studies should explore the effect of other time changing variables (HAART access policy, OI treatment/ prophylaxis, adherence, drug resistance, etc.) on the trends. Projections using alternative models such as ARIMA models without a constant term can also be explored in the future. Another limitation was that, although the model adequacy was evaluated, ARIMA models can only be used for short term forecasts and the accuracy of the predicted values gets less the longer the prediction period.

Despite the above limitations, the greatest strength of the present study is that it is the first in Uganda to model and predict out-of-sample prevalence of OIs among HIV positive patients on HAART. However, additional studies from other ART programmes in the country are needed in order to generalize the results at the national level.

## Conclusion

While the prevalence of any OI among HIV positive individuals on HAART in Uganda is expected to decrease overall, it’s unlikely that OIs will be completely eliminated in the foreseeable future. There is urgent need to integrate control/prevention measures for all OIs in HIV/AIDS care programmes and to address all factors that may undermine HAART effect including poverty if OIs are to be eliminated in these settings. The forecast model in the current study could be the first attempt to predict morbidity due to OIs among HIV positive individuals on HAART in Uganda. However, ARIMA models are designed for short term forecasts. Future observational series should explore other predictive models that provide longer forecasts taking into account time changing variables and random disturbances in the time series.

## Abbreviations

ACF, autocorrelation function; AIC, Akaike information criteria; AIDS, acquired immuno-deficiency syndrome; AR, autoregressive; ARIMA, autoregressive integrated moving average; ART, antiretroviral therapy; ARV, antiretroviral; BIC, Bayesian information criteria; HAART, highly active antiretroviral therapy; HIV, human immunodeficiency virus; IQR, interquartile range; IRB, institutional review board; MA, moving average; MAPE, mean absolute percentage error; OI, opportunistic infection; PA F, patial autocorrelation function; SD, standard deviation; TASO, the AIDS support organisation; UNAIDS, joint United Nations programme on HIV/AIDS; WHO, World Health Organisation

## Additional file

Additional file 1: Data on monthly prevalence of any opportunistic infection among HIV positive individuals on HAART in TASO, Uganda (2004-2013). (XLS 34 kb)
